# A 181 GOPS AKAZE Accelerator Employing Discrete-Time Cellular Neural Networks for Real-Time Feature Extraction

**DOI:** 10.3390/s150922509

**Published:** 2015-09-04

**Authors:** Guangli Jiang, Leibo Liu, Wenping Zhu, Shouyi Yin, Shaojun Wei

**Affiliations:** Institute of Microelectronics, Tsinghua University, Beijing 100084, China; E-Mails: jglwiz@gmail.com (G.J.); zhuwp08@mails.tsinghua.edu.cn (W.Z.); yinsy@tsinghua.edu.cn (S.Y.); wsj@tsinghua.edu.cn (S.W.)

**Keywords:** AKAZE, binary feature descriptor, feature extraction, hardware architecture, VLSI implementation

## Abstract

This paper proposes a real-time feature extraction VLSI architecture for high-resolution images based on the accelerated KAZE algorithm. Firstly, a new system architecture is proposed. It increases the system throughput, provides flexibility in image resolution, and offers trade-offs between speed and scaling robustness. The architecture consists of a two-dimensional pipeline array that fully utilizes computational similarities in octaves. Secondly, a substructure (block-serial discrete-time cellular neural network) that can realize a nonlinear filter is proposed. This structure decreases the memory demand through the removal of data dependency. Thirdly, a hardware-friendly descriptor is introduced in order to overcome the hardware design bottleneck through the polar sample pattern; a simplified method to realize rotation invariance is also presented. Finally, the proposed architecture is designed in TSMC 65 nm CMOS technology. The experimental results show a performance of 127 fps in full HD resolution at 200 MHz frequency. The peak performance reaches 181 GOPS and the throughput is double the speed of other state-of-the-art architectures.

## 1. Introduction

Recently, visual sensor networks (VSNs) have attracted considerable attention in the research field. They are able to collect, process and communicate visual information in their surrounding environments [1]. They are already available in a wide range of visual applications, such as environmental monitoring [2] and virtual reality [3]. Real-time visual feature extraction is a key requirement for VSNs. The visual feature is a fundamental algorithm that is widely used in many high-level visual applications, such as augmented reality and object recognition [4,5,6,7,8,9]. Furthermore, the local processing of image data can significantly reduce the communication burden. Complex vision analysis can also provide VSNs with high levels of intelligence. These algorithms (such as feature extraction) enable VSNs to collaborate by exchanging detected features and collectively estimating the behavior of the captured object [10].

It is difficult to directly embed and locally process in real time for applications on VSNs because the feature extraction algorithm is computationally intensive [11]. Numerous efforts have been made to accelerate these vision algorithms by VLSI implementation. Huang *et al.* implemented SIFT [12] on a parallel architecture [13] that separately used interactive components for detector and descriptor tasks. The accelerator reached 30 fps for VGA images at 100 MHz frequency. In this system, the descriptor generation module accounted for 89% of the total time and became the bottleneck. Chiu *et al.* proposed a layer parallel SIFT feature and implemented it on a hardware architecture [14]. The design used an integral image technique to accelerate the scale pyramid build. The proposed system achieved 30 fps for images with different resolutions. As the image size increased, the number of descriptors was reduced due to the limited memory bandwidth. More recent studies have paid closer attention to the acceleration of the descriptor part. Jiang *et al.* introduced a real-time SIFT accelerator with a task-level parallel and pipeline technique [15]. In this design, a window dividing method was proposed to avoid sample patch rotation in the descriptor. This technique reduced computational complexity and gained a 15× speed increase for descriptor generation. The system processed a 512 × 512 image in 6.55 ms. Other hardware designs are based on binary descriptors that show significant efficiency improvements. In [11], Wang *et al.* proposed a real-time FPGA-based embedded system architecture that employed a SIFT detector and BRIEF [16] descriptor. This architecture achieved 60 fps for 720 p video. In general, most of the current designs are based on traditional features, such as SIFT or SURF. These features employ float descriptors that may cause a communication burden for the VSNs [17]. For designs based on binary descriptors, high throughput was realized at the expense of lower robustness in some aspects of the transformation due to the BRIEF descriptor [11,18].

In order to achieve real-time performance, the design for the current research is based on the accelerated KAZE (AKAZE) feature [19]. The AKAZE feature employs nonlinear scale space and a binary descriptor, which provides a considerable trade-off between speed and accuracy. Although AKAZE embeds a recent numerical scheme in order to accelerate the scale pyramid build, the computational complexity still poses a challenge for the hardware design. Also, considerable memory burden still exists due to the data dependency in the pyramid build and descriptor generation.

In light of the above considerations, this paper proposes a flexible embedded system architecture for real-time feature extraction. The architecture embeds a different kind of block-serial scheme in order to reduce the hardware cost and a pixel-level parallel scheme to increase throughput. It achieves a high throughput while maintaining a comparable performance to the original AKAZE algorithm. The contributions of this work are as follows:

- The authors believe this to be the first feature extraction design based on the AKAZE algorithm. The AKAZE feature was mapped to an octave-serial architecture (OSA) primarily consisting of a two-dimensional pipeline array. It decreases the hardware resource requirement and also provides sufficient flexibility for the various application fields, characterized as different image resolutions, precision and power consumption.
- A substructure consisting of a block-wise discrete-time cellular neural network (B-DTCNN) is presented. It decreases memory demand through the reduction in data dependency.
- A hardware-friendly descriptor, termed the robust polar binary descriptor (RPB), is presented. The polar arrangement of the sample pattern, combined with a simplified technique to realize rotation invariance, greatly decreases the memory burden and computational complexity.

Section 2 analyzes the AKAZE algorithm in order to determine possible obstacles. It also introduces the hardware-friendly descriptor, RPB. Section 3 presents the hardware design details. Section 4 presents the experimental and simulation results. Section 5 provides the conclusions.

## 2. Algorithm Optimization

This section provides a brief introduction to the AKAZE feature. Further analysis demonstrates the advantages and disadvantages of AKAZE to achieve real-time performance for hardware solutions. Finally, a hardware-friendly binary descriptor is introduced to reduce memory cost and computational complexity.

### 2.1. AKAZE Overview

The AKAZE feature contains three major stages: nonlinear pyramid build, key point location and binary descriptor generation. In the first stage, AKAZE employs the Perona-Malik (P.M.) equation [20] to build a nonlinear scale pyramid. In order to construct different sublevels in the pyramid, this method diffuses the original image to a series of increasing scale levels using Equation (1), where *I* is the image luminance and *k* is the contrast factor. In order to accelerate the diffusion process, AKAZE adopts a fast explicit diffusion (FED) scheme [20,21] that approximates the solutions by iterations. Each iteration can diffuse the image with a small-scale step. Based on variable scale-steps (rather than constant), FED greatly reduces the number of iterations:

(1) $$\frac{\partial I}{\partial t}=div(\frac{\nabla I}{1+|\nabla I{|}^{2}/k^{2}})$$

The key points are located in the second stage. Once the pyramid is constructed, the determinant of the normalized Hessian matrix is computed (Equation (2)). Next, the local maxima at each sublevel are picked out as candidate key points. In order to search the scale extremes, a potential point is compared with other candidate key points within a σ × σ window from sublevel i − 1 to i + 1:

(2) $$H_{norm}=\sigma^{2}\left|\begin{array}{cc} I_{xx} & I_{xy}\\ I_{xy} & I_{yy} \end{array}\right|$$

The third stage of AKAZE introduces a modified-local difference binary (MLDB) descriptor. Typically, MLDB descriptor generation is divided into three steps: main orientation estimation, sample patch rotation and binary descriptor generation. Firstly, MLDB estimates the main orientation using a SURF-like method based on the histogram. Then, the sample pattern is rotated according to the direction of the key point. Finally, the binary descriptors are generated through comparison among the grids in three channels (one luminance and two rotated first-order derivatives).

### 2.2. AKAZE Analysis

**Figure 1:**
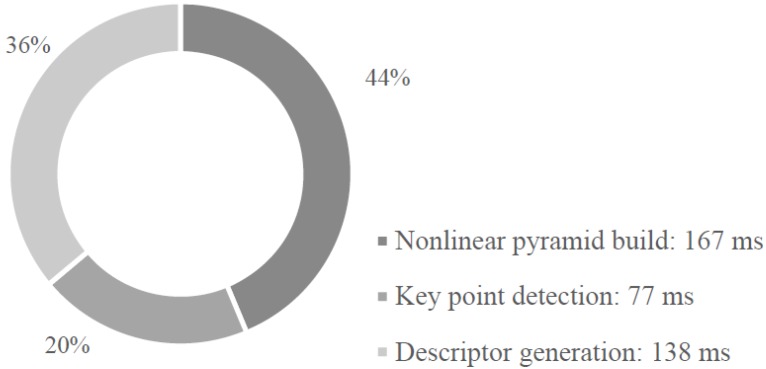
Runtime analysis of AKAZE on PC platform (Intel i5-3210M @2.5 GHz).

Brief runtime analysis of AKAZE is conducted on a PC platform (Intel i5-3210M @2.5 GHz) in order to optimize the configuration. A “boat” image from the Oxford dataset [22] is used for the AKAZE algorithm testing. Figure 1 shows the time usage of each stage. The first stage (nonlinear pyramid build) is the most time-consuming component, taking approximately 40% of the total time. The third stage requires 0.034 ms to calculate a descriptor. The PC platform can only achieve 3 fps and struggles to meet real-time performance for high-resolution images.

**Figure 2:**
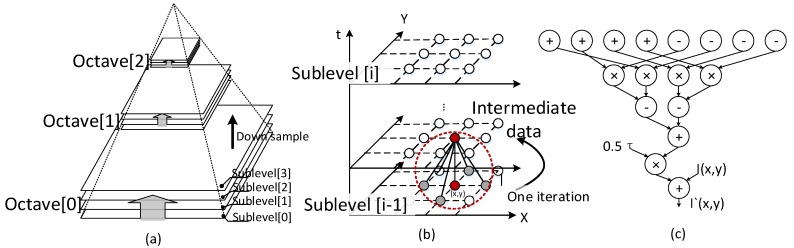
Nonlinear scale pyramid build process: (**a**) scale pyramid build process; (**b**) process to generate next sublevel (various iterations); (**c**) operations to update one pixel within an iteration.

Further analysis of AKAZE shows one main advantage and two significant disadvantages for the hardware design. As an advantage, AKAZE provides a novel simplified method to find spatial and scale extremes. It divides the process into two different steps and introduces candidate key points as intermediate data. The memory demand can be reduced because there is no need to align data for scale extreme searching in the adjacent sublevels. However, there are still two significant challenges for achieving real-time performance. The first challenge is a nonlinear pyramid build. Several nonlinear filters must be processed in series in order to build four sublevels within one octave (Figure 2a). A nonlinear filter contains various iterations (Figure 2b), each having 17 arithmetic operations (Figure 2c). Due to the generation process of the cascade, the increase of sublevels and octaves may lead to unacceptable hardware resource usage under the current architecture design [11,13,23]. Also, the frame-level intermediate data in a nonlinear filter generate large memory demand.

The second challenge is the descriptor generation. The AKAZE uses a traditional 3-stage process flow. Similar hardware designs [13,14,24] indicate that this design is a bottleneck for system throughput. Also, it poses challenges for hardware resources (especially memory demand). Firstly, the pixels around a key point are read out in the first and third stages. This not only encumbers the algorithm pipelining in the hardware, but also causes significant random memory access.

Recent studies [11,15,23] show that random access is the main barrier to achieving real-time performance. This challenge is difficult to resolve only using hardware design. Also, the data random access leads to excessive buffer caching for the intermediate data around a key point. Additionally, for rotation invariance, the coordinates (*x*, *y*) need to be rotated according to the main direction, θ, in the second stage (Equation (3)) as well as the first-order derivative in the third stage, which results in numerous triangle functions:

(3) $$\left[\begin{array}{l} x^{\prime }\\ y^{\prime } \end{array}\right]=\left[\begin{array}{cc} cos\theta & -sin\theta \\ sin\theta & cos\theta \end{array}\right]\left[\begin{array}{c} x\\ y \end{array}\right]$$

### 2.3. AKAZE Optimization

In this section, a hardware-friendly binary descriptor is proposed. It aims to overcome the memory burden challenge in the hardware. The challenge results from the number and order of random access, which is difficult to overcome only using hardware design. The PRB descriptor is introduced in order to find a method to speed up generation. It consists of a polar sample pattern combined with local integral images. It successfully converts most random access into regular access. Also, a simplified method to estimate the main orientation is proposed based on the polar sample pattern. It further reduces random memory access as well as computational complexity. In addition, the PRB descriptor easily pipelines in hardware because it only accesses the integral image once. Details of the RPB descriptor are as follows.

Descriptor generation is always the bottleneck in hardware design. This general process can be divided into three stages: main orientation estimation, patch rotation and local descriptor generation. Analysis shows that the process contains significant random memory access in the first and third stage, which poses a challenge to achieve high throughput (see Section 2.2). Also, the random scatter of key points brings extra complexity to the control. Huang [13] proposed an interactive architecture with a co-processor for descriptor generation that successfully overcame the control complexity of the random distribution. However, 89.7% of the time was used by the descriptor generation. Several hardware solutions [11,13,15] were proposed to accelerate the descriptor generation. Although substantial improvements were achieved, the processing speed was still unable to match key point detection. As such, the current research introduces a hardware-friendly descriptor.

**Figure 3:**
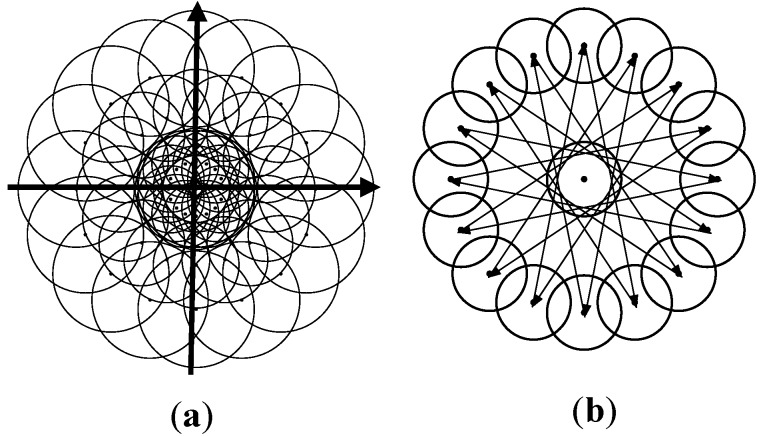
(**a**) Sample pattern containing three layers, where each layer has 17 sample grids with polar arrangement; (**b**) Pairs used for main orientation estimation based on the outermost layer.

The RPB descriptor has three major stages. Firstly, according to the sample pattern, it reads out all the grid values for later use. It contains two channels: the grey value and the gradient norm. The main orientation is estimated using the grid values of the outermost layer in the first channel. Secondly, invariance to rotation is achieved by reordering the sample values according to the main direction. Thirdly, pseudo-random pairs are selected for comparison. The two key features that focus on simplification of rotation invariance are as follows:

*Sample Pattern*: The RPB descriptor employs a novel sample pattern of polar arrangement for rotation invariance through reordering and less random memory access. Figure 3a shows the three layers of the sample pattern. The arrangement of each layer is similar to DAISY [25] and has various radiuses and distances from the center point. For each layer, there are sixteen grids on the circle that matches the number of discrete angles for reordering.

*Main Orientation*: The RPB descriptor adopts a simplified gradient method similar to BRISK [26] for rotation invariance. Equation (4) shows that the angle vector, *A*, is estimated through the sum of gradients of specific pairs in the set *P*. The two-dimensional vector, *p*, contains the grid coordinates and *I*(*p*) is the grey value of the first channel. The sixteen selected pairs are symmetrically distributed to avoid a division operation (Figure 3b):

(4) $$A=\sum_{(p_{i},p_{j})\in P}\left(p_{i}-p_{j}\right)\left[I(p_{i})-I(p_{j})\right]$$

Based on the hardware-oriented optimization, the RPB descriptor reduces both the memory burden and computational complexity. In terms of memory usage, random access decreases by 68.7% (650 to 240 per key point). Based on the integral image, the polar sample pattern changes most of the random access into regular access. In addition, the proposed main orientation estimation method does not add extra access to the integral image memory, thereby removing approximately 40% of the memory burden. Also, the optimized method to estimate the main orientation significantly decreases computations, especially in the triangle computation (Table 1). For robustness, the RPB descriptor achieves similar matching accuracy compared with the original algorithm; however, accuracy is slightly less in the rotation and brightness tests (See Section 4.1).

**Table 1:** Comparison of operations to estimate main orientation.

Operations	Proposed	Original (SURF-Like)	BRISK (OpenCV)
Extra Random Access	0	218	240
Trigonometric Function	1	452	1
Addition/Subtraction	80	1746	4350
Multiplication	32	452	1741
Lookup Tables	32	109	3480

## 3. Proposed Hardware Architecture

**Figure 4:**
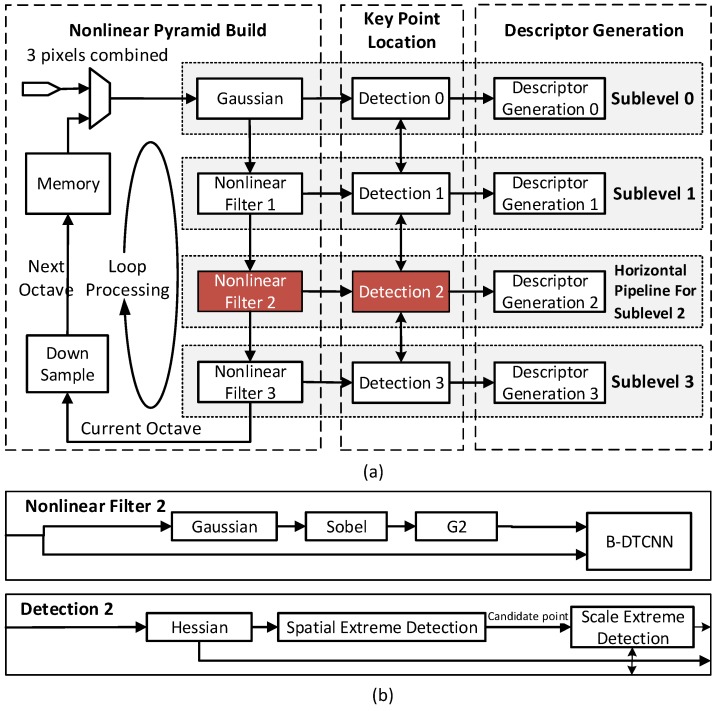
(**a**) Proposed OSA architecture with three main stages due to the sublevels’ process in pyramid; the different octaves are processed in series with various pixels processed in parallel in each module for efficiency; (**b**) Typical block diagrams of a nonlinear filter module and detection module used to illustrate timing design.

This section introduces details of the hardware architecture based on the optimized AKAZE feature. The proposed system consists of three main functional parts (Figure 4). In order to achieve high throughput with a reasonable hardware cost, three techniques (OSA, B-DTCNN and PRB) are utilized in the design.

### 3.1. Octave-Serial System Architecture

This section introduces the OSA system architecture (Figure 4). A two-dimensional pipeline array is applied to make full use of computational similarities among the different octaves. The OSA architecture is able to reduce hardware resources as well as provide additional flexibility to image size and speed-precision tradeoffs in real-time.

The number of octaves directly affects matching accuracy. However, multiple octaves result in extra hardware costs. In [11,13,23], similar functional modules, such as key point location and the descriptor generation module, are repeated in parallel to process data in the second or third octave. The current analysis shows that multiple octaves does not significantly increase throughput. For example, the second, third and fourth octave only add 25%, 6.25% and 1.56% extra pixels, respectively. Therefore, processing octaves in series significantly reduces hardware costs with only a minimal sacrifice in speed.

**Figure 5:**
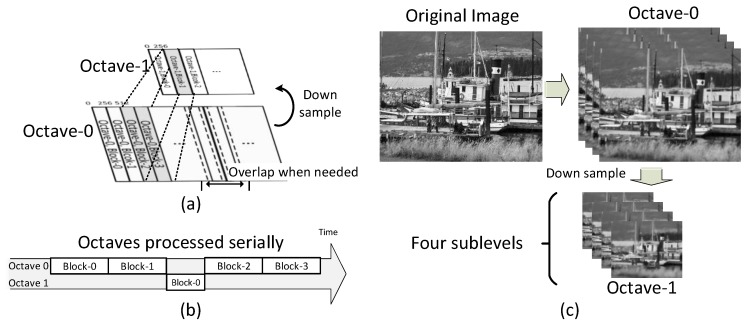
(**a**) Block-partitioning scheme in a nonlinear scale pyramid; (**b**) Specific order to process octaves in series, where different blocks in multi-octaves are divided and combined in a ribbon-like form to fit the octave-serial system architecture; (**c**) An example of a two-octave pyramid.

Figure 4a depicts the whole OSA architecture, which can be considered as a two-dimensional pipeline array. The vertical pipeline constructs a scale pyramid through cascaded nonlinear filters. It continuously reads in the stream of the original or down-sampled image and sends four sublevels within one octave to the next stages. There are four horizontal pipelines and each processes one sublevel: (1) locate key points based on the Hessian response of adjacent levels, which sequentially searches space and scale extremes; (2) generate descriptors depending on the local integral image. Figure 5 shows the processing flow. Unlike the general block-wise scheme, this block-partitioning scheme penetrates into the inner scale pyramid. The image data of the different octaves are divided into several trunks (Figure 5a) and combined in a ribbon-like form for processing (Figure 5b). The accelerator moves to process one chunk in the next octave (in the memory) after finishing two chunks in the current octave. In this way, the proposed system processes the data of different octaves in series on the same hardware. Figure 5c is an example of a two-octave pyramid in which there are four sublevels within one octave and each sublevel is processed by one horizontal pipeline. Also, overlap between the adjacent chunks can be added. Furthermore, the off-chip memory can cache one more down-sampled trunk if the key points near the block border (approximately 15–20 pixels) are necessary in practice; otherwise, these points are ignored in the descriptor generation stage. The timing sequence is as follows. Most of the modules, such as linear filter (Gaussian, Sobel and Hessian) and detection, are capable of processing three pixels per cycle. However, the B-DTCNN and descriptor modules are bottlenecks in the structure. In the current design, the B-DTCNN processing ability is 1.64 pixels per cycle, which determines the system throughput (see Section 3.2). For each descriptor module, 51 cycles are required to generate a descriptor. Through the buffer and discarding schemes, the descriptor module does not stall the pipeline (see Section 3.3). Due to the loop-processing scheme, the whole system can run at 127 (200 M × 1.64/(1920 × 1080 × 1.25)) fps in full HD, with two octaves in pyramid.

The OSA architecture has two main advantages. Firstly, an OSA-based system provides considerable flexibility in image size, precision and power consumption. Due to the block-wise method, the system proposed can easily process images in different resolutions such as 720 p, 1080 p and 4 K. The OSA-based system is more robust to image scale change due to the handling of multiple octaves. The power consumption can be reduced by approximately 25% when shutting off the last horizontal pipeline. Secondly, having a similar hardware cost, the system provides an extra 54% throughput compared with previous works [11,13,24]. Due to the change from octave-parallel order to octave-serial order, the similar hardware cost provides a higher degree of pixel-level parallelism within one octave. When the pixel-parallelism degree doubles, the second and third octave only yield 25% and 6.25% extra pixels processing, respectively (due to the octave-serial order). Hence, the processing speed sharply increases. Moreover, due to its design flexibility, other feature extraction algorithms, such as SIFT, can be mapped to the proposed architecture. The architecture also provides efficiency and flexibility to algorithms that use linear filters to construct pyramid. Although the OSA architecture greatly accelerates key point detection, the throughput is still limited by the descriptor generation module and the nonlinear filter module. These challenges are addressed in the following sections.

### 3.2. Block-Serial DTCNN

This subsection presents a substructure named B-DTCNN that significantly decreases memory demands brought about by the nonlinear pyramid construction. An analysis of the relationship between the Gaussian convolution and nonlinear diffusion provides a block-wise serial strategy. The strategy adopts elastic overlap between blocks in order to gain a better trade-off between accuracy and hardware costs. Finally, the corresponding hardware structure is presented, which contains a Ping-Pong structure to accelerate throughput.

The DTCNN architecture [27] is used to construct the pyramid because of its local connection and parallelism. Due to frame-level data dependency in iterative filters, the intermediate data cache results in a significant memory demand (see Section 2.2). Therefore, it could only use block level rather than whole image processing. Furthermore, the nonlinear pyramid requires a series of images at different run-time stages. In [28], a sequential DTCNN architecture was proposed based on FPGA. The computing task of pixels in an iteration was mapped to one cell where the images at different run-times could be easily passed to the next stage. However, the buffers between the different stages resulted in long latency and memory costs. Inspired by the above design, this paper proposes a B-DTCNN, in which pixels within a block are processed in parallel.

The removal of frame-level dependency is based on the following analysis. When the flow function, *g*, becomes a constant function, an anisotropic diffusion Equation (5) evolves to linear diffusion Equation (6) and the nonlinear scale pyramid becomes a Gaussian scale pyramid:

(5) $$\frac{\partial I}{\partial t}=div(g(|\nabla I|)\cdot \nabla I)$$

(6) $$\frac{\partial I}{\partial t}=div(\nabla I)$$

The linear diffusion equation can be solved two ways. One way is the Gaussian template convolution with the original images, where the evolution time, *t*, is mapped to the scale parameter, σ, in Equation (7). The other way is a numerical scheme identical to anisotropic diffusion (such as an explicit scheme). The first solver shows that the longer the distance between the pixel and center, the less it contributes to the result; the corresponding pixels have little impact when the distance is more than a given threshold. Moreover, the threshold distance is proportional to the evolution time, *e*, in each stage:

(7) $$t_{i}=\frac{1}{2}\sigma_{i}^{2},\;i=0,1,2...$$

**Figure 6:**
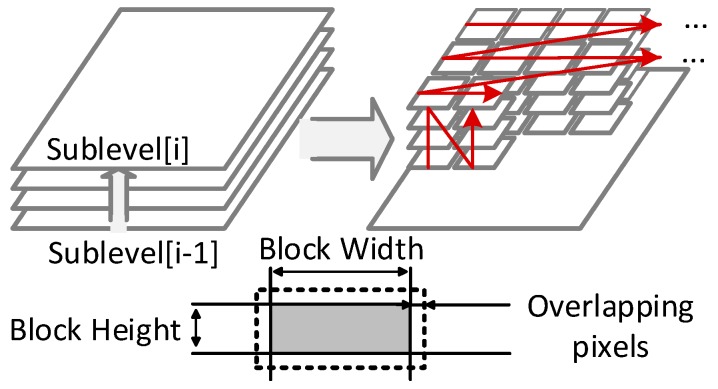
A block-serial scheme in B-DTCNN where the frame-level data dependency is deconstructed to block-level one. The blocks of different stages choose elastic overlapping pixels due to time steps for different trade-offs between accuracy and hardware costs because the block configure determines the size of the PE arrays.

The block layout (Figure 6) has three key parameters: overlapping pixels, *O*, which directly affect diffusing accuracy; block width, *W*; and block height, *H*, all of which determine the hardware cost and system throughput. In general, the mean square error (MSE) is used to evaluate the difference between the original pyramid, *I*, and the optimized *I*′(O, W, H).

(8) Diffusing Distortion:MSE(I, I′(O,W,H))

**Figure 7:**
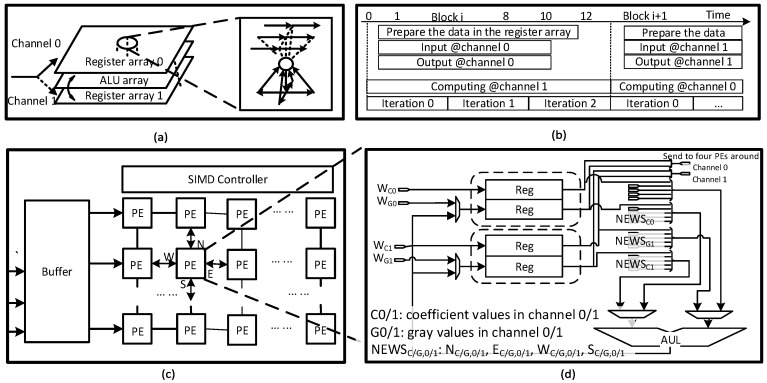
(**a**) Data flow of the Ping-Pong operations. The arithmetic operations and data preparation are done in parallel; (**b**) Typical timing sequence to process one block; (**c**) Diagrams of B-DTCNN array in which PEs are connected in North, South, East and West (NEWS) directions to communicate data during iterations; (**d**) Inner structure of each PE that caches coefficient values and gray values of two channels in local registers.

Figure 7 shows data flow and hardware implementation. From the view of PE array, two symmetric register arrays are used to construct a Ping-Pong structure (Figure 7a). In each iteration, the ALU array executes the arithmetic operation and exchanges the internal temporary data with one register array when the other register array switches data with the external modules. Inside a PE, there are four registers to cache grey values and nonlinear coefficients of two channels separately and to communicate data with PEs in North, South, East and West (NEWS) directions (Figure 7d). Each iteration requires four cycles. After several iterations, the block of data is sent to the next stage for further processing. The speed of the nonlinear filter block is determined by the number of iterations and the size of the processing array, which further determines the throughput of the vertical pipeline. For the timing consideration, Figure 7b shows an example of the B-DTCNN module in the first nonlinear filter (5 × 9 array). Due to the diffusion parameter, it only has one overlapping pixel. Therefore, it requires 12 cycles (3 iteration) and outputs 21 (3 × 7) pixels. The speed is 1.75 (21/12) pixels/cycle. For each row, “padding” pixels are used on the border and idle cycles are used to synchronize the other two nonlinear filters. Hence, the unified processing speed is 1.64 pixels/cycle.

**Figure 8:**
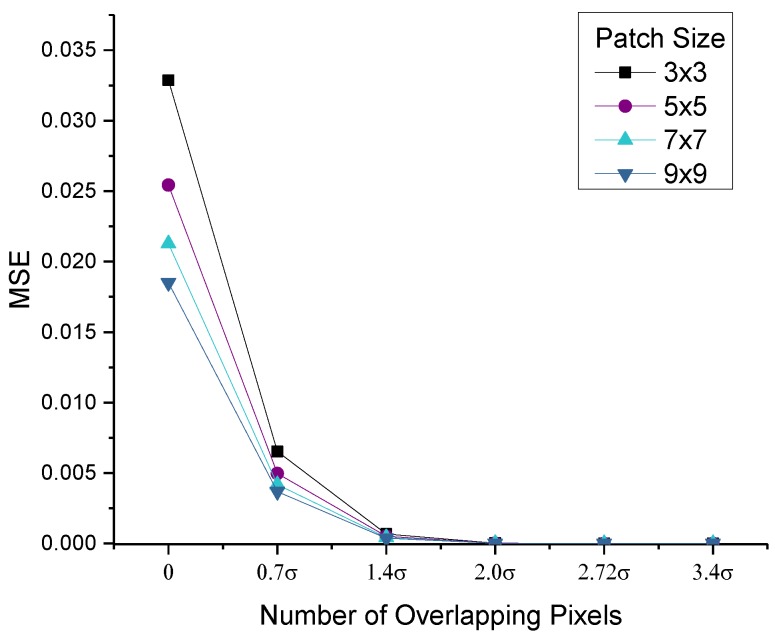
Error between the diffused images using two kinds of iterations (full-frame iteration and independent block-level iteration). The error quickly drops with the increase of overlapping pixels.

The pyramid distortion dramatically decreases when the overlapping pixels increase (Figure 8). In general, the overlapping pixels are set to more than 2σ to minimize the error (in the order of 10^−5^), which conforms to the solver of the Gaussian convolution (where the radius of the Gaussian template is always 2σ ~ 3σ). In the current hardware design, the memory demand decreases by approximately 80% compared with the design in [28]; furthermore, there is only 13.7% redundant pixel computation in the image boundary.

### 3.3. Robust Polar Binary Descriptor Module

This subsection introduces the descriptor generation module. The structure is based on the proposed hardware-friendly RPB binary descriptor. The hardware/software co-design successfully reduces the random memory access number and adjusts the access order. Hence, the RPB descriptor can be mapped to a three-stage pipeline. Finally, this module is able to generate the descriptor at relatively high speeds.

**Figure 9:**
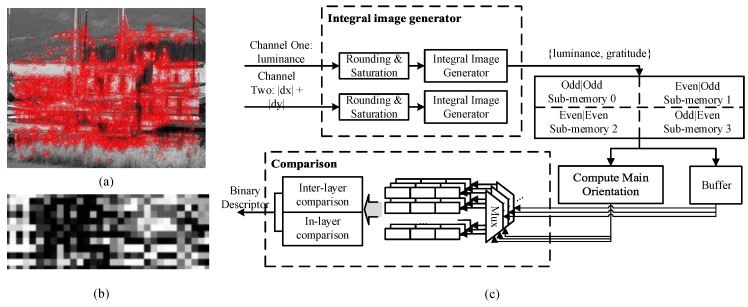
(**a**) An example of the input sublevel with key points detected; (**b**) An example of the binary descriptors in which each grid represents eight binary bits; (**c**) Block diagram of descriptor generation module containing three main stages: integral image build, grid value read-out with main orientation estimation, and grid values comparison.

Figure 9c shows that the descriptor generation module primarily consists of a three-stage pipeline in the hardware. Firstly, two channels of one sublevel (Figure 9a) are used to construct the integral image and both results are combined. This operations reduce the memory random access. The memory block consists of four partitions to further speed up the random access, especially for the integral image [23]. Also, the circular data dependency around a key point results in significant memory demand. Therefore, the integral images are compressed. One way is through module-N arithmetic based on [29]. The other way is through general rounding and saturation operations with minimal accuracy loss. Secondly, all the grid values are read out and then buffered in cache. The main orientation is estimated based on the first sixteen grid values. Thirdly, the rotation invariance is achieved by reordering the sixteen grid values in each layer. Then, comparisons are performed in order to generate the final binary descriptor (Figure 9c). The following analysis shows that the proposed structure has strong robustness against random memory scatter access. The whole process can be treated as a three-stage pipeline. The most time-consuming stage is to read the sample grid from memory. The operation requires 51 cycles. The average speed to feed a row in memory for an integral image is 156 cycles. The key points are abandoned when the number of points exceeds 10 in three continuous rows (each containing 256 pixels) within one sublevel. According to the test on the Oxford datasets, the extreme case does not occur. Within one sublevel, the average number of key points in each row is less than one. Therefore, the descriptor generation module speed matches the detection speed in most cases.

Due to the co-design of hardware and software, the descriptor generation module achieves real-time performance. The optimized method to estimate the main orientation enables the process to be pipelined. The memory burden is reduced by the integral image and the novel sample pattern. The random access is further accelerated by the embedded memory partition scheme for the integral images. For the whole architecture with four modules, the peak generation speed is 12.5 cycles per key point, which matches the detection speed in most cases.

## 4. Simulation and Verification

In this section, the performance of the optimized AKAZE is tested using detector repeatability and matching accuracy metrics. The hardware implementation details are also presented (focusing on system throughput and hardware cost).

### 4.1. Feature Accuracy

Firstly, a brief introduction to the test configuration is provided. The pyramid parameter is selected due to the flexibility with octaves that result from the proposed architecture. There are two octaves, each with four sublevels. In the benchmark, fixed-point precision is adopted in the optimized AKAZE with various bit widths in the different stages (while maintaining the original floating-point precision in the original one). In order to assess the accuracy of the different transformations, image sets from the Oxford datasets are adopted for algorithm evaluation. The benchmark criteria is introduced below. The repeatability [30] is selected to measure the ability of the detector to extract repeatable key points. The overlap error is less than 0.4. A third-part framework [31], used in [32], is applied to evaluate the descriptor. In the benchmark, matching accuracy is mainly used to show the robust quality of the key points, which combines the detector and descriptor.

**Figure 10:**
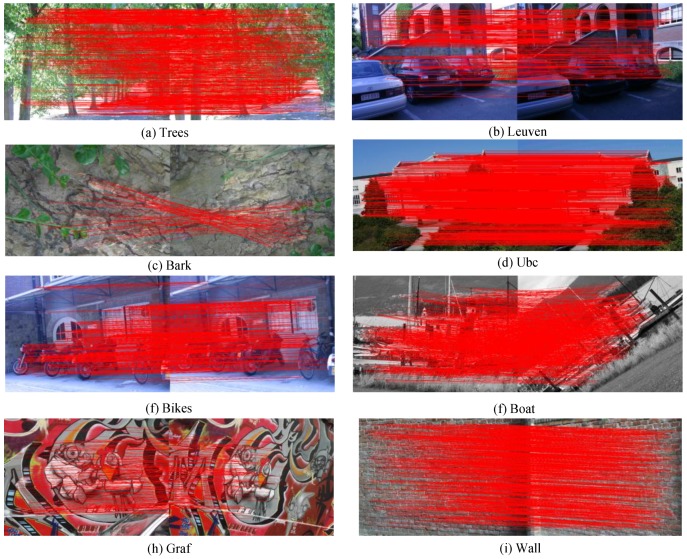
Feature matching results.

**Figure 11:**
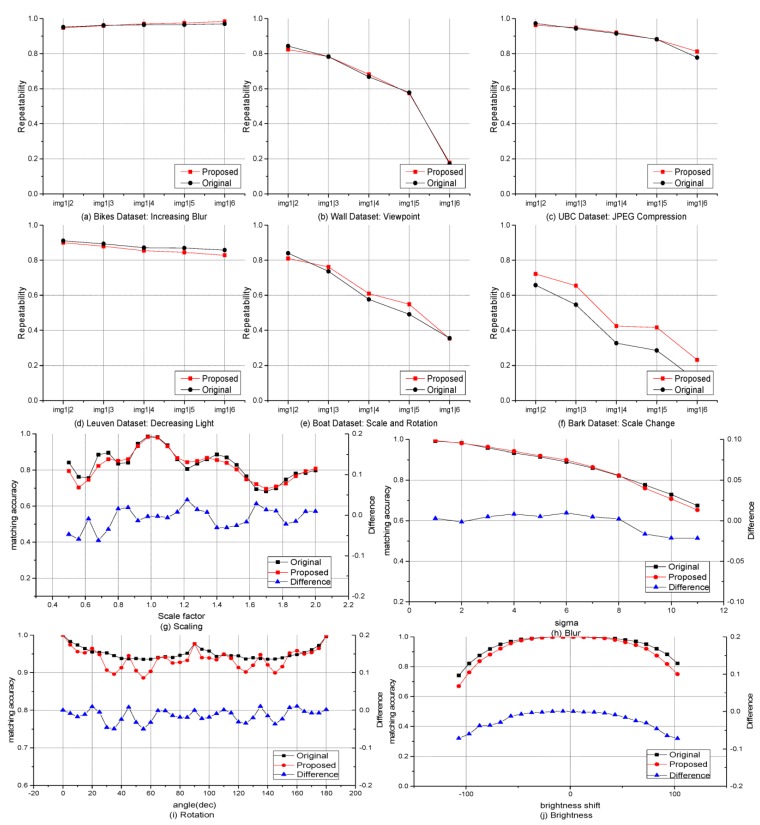
Comparison of detector repeatability (**a**–**f**) and matching accuracy (**g**–**j**).

Figure 10 shows the feature-matching results that more intuitively demonstrate the simulation results. Quantitative results are provided for comparison (Figure 11 and Table 2). The results show that the optimized algorithm obtains similar performance compared with the original AKAZE algorithm. Table 2 indicates that, on average, the matching accuracy of the proposed feature only marginally drops (approximately 1%–2%). Figure 11 also shows these results. The matching accuracy in the rotation change drops a little at the boundary of each discrete angle for the proposed RPB descriptor (Figure 11i). Also, there are significant changes in brightness when the accuracy declines (Figure 11j) because of the constant contrast factor, *k*, in Equation (1) rather than an adaptive one as in the original AKAZE. The scaling change improvement can be attributed to the adjustment of the scale parameter in the second octave. The chosen scale parameter is similar to SIFT.

**Table 2:** Comparison of average matching accuracy.

		Brightness	Gaussian	Viewpoint	Rotation	Scaling
**Boat**	AKAZE	87.47%	93.29%	78.53%	95.51%	83.03%
	Proposed	83.70%	93.78%	78.95%	93.99%	82.30%
**Trees**	AKAZE	88.97%	89.45%	72.66%	90.74%	80.26%
	Proposed	88.07%	92.77%	76.75%	92.57%	82.55%
**Bikes**	AKAZE	90.03%	95.34%	77.17%	91.01%	82.89%
	Proposed	88.03%	94.69%	76.81%	88.32%	81.76%
**Bark**	AKAZE	93.24%	95.92%	76.80%	92.14%	86.18%
	Proposed	90.88%	95.20%	76.53%	93.66%	84.37%
**Graf**	AKAZE	88.37%	95.96%	77.92%	93.23%	85.00%
	Proposed	84.06%	95.64%	77.65%	91.01%	82.69%
**Leuven**	AKAZE	90.80%	96.36%	78.73%	89.27%	84.83%
	Proposed	89.21%	94.86%	78.86%	85.50%	82.58%
**Ubc**	AKAZE	89.43%	93.25%	79.24%	94.43%	82.33%
	Proposed	88.25%	94.28%	81.48%	91.52%	83.68%
**Wall**	AKAZE	87.98%	89.06%	77.64%	93.05%	82.81%
	Proposed	85.09%	91.68%	76.48%	95.09%	83.58%

### 4.2. Hardware Performance

**Figure 12:**
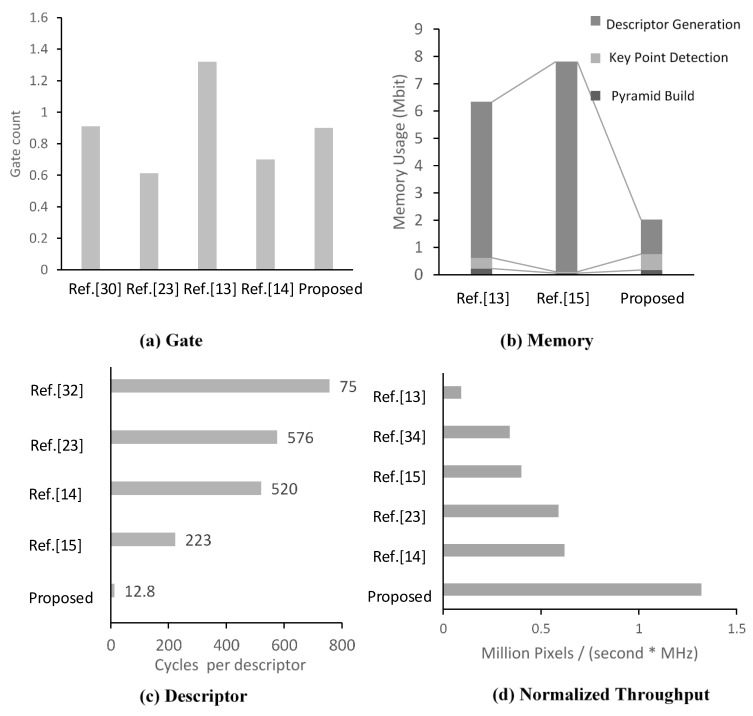
Comparison of hardware cost (**a**,**b**) and process speed (**c**,**d**).

**Figure 13:**
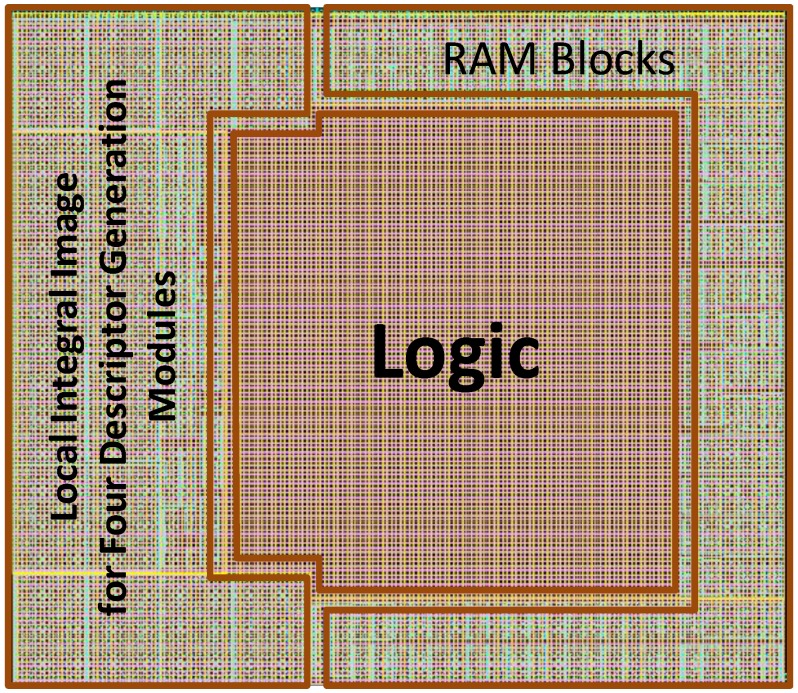
Layout of the proposed AKAZE accelerator.

The proposed architecture is mapped to hardware using TSMC 65 nm CMOS technology. Due to lack of AKAZE-based hardware designs, the current design is mainly compared with state-of-the-art works based on SIFT or SURF, which have similar workflows (see Table 3). Figure 12 shows a comparison of hardware costs. Figure 13 shows the layout of the die, which is generated by the integrated circuit compiler (ICC). Table 4 provides a summary of the hardware resources.

The multiplication and addition are taken into account for the measurement of peak performance [33]. For the linear filter module, there are 3*n* − 1 fixed-point operations per pixel, where *n* is the template size. For the B-DTCNN module, there are 17 operations/pixel for every iteration. Each descriptor generation module is capable of calculating three key points per row. Hence, the speed to process a key point is 0.047 (4 × 3/256) key points per pixel. For each key point, there are 31 operations to estimate the main orientation and 306 operations to construct the sample grid. The operations required to generate descriptors are 20 (2 + 0.047 × (306 + 31)) operations/pixel. Table 5 provides a summary of the number of operations in each stage. In total, the whole structure reaches 181 (127 × 1920 × 1080 × 1.25 × 550) GOPS. In order to compare the processing speed, the throughput is normalized to the unit frequency and the equivalent gate in which the memory is converted to gate according to TSMC 65 nm technology. Figure 12d and Table 3 show that the proposed system is nearly twice as fast as comparable state-of-the-art works. Figure 12c shows that the high throughput can be attributed to the proposed RPB descriptor, which is no longer the bottleneck in the hardware.

**Table 3:** Comparison of performance.

	Reference [23]	Reference [34]	Reference [13]	Reference [14]	Reference [15]	Proposed	Proposed
Approach	SURF	SURF	SIFT	SIFT	SIFT	AKAZE	AKAZE
Pyramid ^1^	(3, 4)	(1, 5)	(3, 6)	(2, 4)	(2, 4)	(2, 4)	(3, 4)
Platform	ASIC	ASIC	ASIC	ASIC	FPGA	ASIC	AISC
Frequency	200 MHz	27 MHz	100 MHz	100 MHz	100 MHz	200 MHz	200 MHz
Memory	3.2 Mb	-	5.73 Mb	0.55 Mb	7.8 Mb	2.12 Mb	2.12 Mb
Gate	0.6 M	-	1.32 M	0.7 M	-	0.95 M	0.95 M
Resolution	1920 × 1080	640 × 480	640 × 480	1920 × 1080	512 × 512	1920 × 1080	1920 × 1080
Speed	57 fps	30 fps	30 fps	30 fps	153 fps	127 fps	121 fps
Throughput	0.118 G	0.0092 G	0.0092 G	0.062 G	0.040 G	0.263 G	0.251 G
Throughput per gate ^2^ (Pixels/s@1 MGate)	31.2 M	-	1.31 M	49.8 M	-	85.9 M	81.8 M
Throughput per frequency (Pixels/s@1 MHz)	0.59 M	0.34 M	0.092 M	0.62 M	0.40 M	1.3 M	1.2 M

^1^ Pyramid parameter is (octaves, sublevels); ^2^ The memory is converted to gate according to TSMC 65 nm CMOS technology.

**Table 4:** Characteristics of the proposed accelerator.

AKAZE Accelerator	
Process	TSMC 65 nm1p10m
Frequency	200 MHz
Gate	0.95 M
Memory	2.12 M bit
Size	4 mm × 3.3 mm
Speed	127 fps (1920 × 1080)

**Table 5:** Operations in each stage.

Stage	Average Operations (Operations/Pixel)
Nonlinear Pyramid Build	290
Key Point Location	240
Descriptor Generation	20
Total	550

## 5. Conclusions

This paper introduces several algorithm and hardware co-design techniques that can realize real-time acceleration for feature extraction. The proposed system maps AKAZE features to a highly flexible system architecture OSA. This provides elastic pyramid parameters and also considerably reduces hardware costs. Two other techniques, B-DTCNN and RPB, are employed to reduce the memory burden, which is the main bottleneck in the hardware design. Based on the above optimizations, the system achieves significantly high throughput for full HD images while maintaining similar accuracy to the original algorithm.
